# Effect of BaF_2_ Variation on Spectroscopic Properties of Tm^3+^ Doped Gallium Tellurite Glasses for Efficient 2.0 μm Laser

**DOI:** 10.3389/fchem.2020.628273

**Published:** 2021-01-08

**Authors:** Jian Yuan, Weichao Wang, Yichen Ye, Tingting Deng, Deqian Ou, Junyang Cheng, Shengjin Yuan, Peng Xiao

**Affiliations:** ^1^Guangdong-Hong Kong-Macao Intelligent Micro-Nano Optoelectronic Technology Joint Laboratory, Foshan University, Foshan, China; ^2^State Key Laboratory of Luminescent Materials and Devices, South China University of Technology, Guangzhou, China

**Keywords:** gallium tellurite glass, Tm^3+^ doped, OH^−^, 1.8 μm emission, BaF_2_

## Abstract

The effects of substitution of BaF_2_ for BaO on physical properties and 1. 8 μm emission have been systematically investigated to improve spectroscopic properties in Tm^3+^ doped gallium tellurite glasses for efficient 2.0 μm fiber laser. It is found that refractive index and density gradually decrease with increasing BaF_2_ content from 0 to 9 mol.%, due to the generation of more non-bridging oxygens. Furthermore, OH^−^ absorption coefficient (α_OH_) reduces monotonically from 3.4 to 2.2 cm^−1^ and thus emission intensity near 1.8 μm in gallium tellurite glass with 9 mol.% BaF_2_ is 1.6 times as large as that without BaF_2_ while the lifetime becomes 1.7 times as long as the one without BaF_2_. Relative energy transfer mechanism is proposed. The maximum emission cross section and gain coefficient at around 1.8 μm of gallium tellurite glass containing 9 mol.% BaF_2_ are 8.8 × 10^−21^ cm^2^ and 3.3 cm^−1^, respectively. These results indicate that Tm^3+^ doped gallium tellurite glasses containing BaF_2_ appear to be an excellent host material for efficient 2.0 μm fiber laser development.

## Introduction

Over the past few decades, fiber lasers operating in eye-safe 2.0 μm spectral region have attracted a great deal of attention due to strong absorption band of several chemical compounds (H_2_O, CO_2_, N_2_O, etc.) in this region (Chen et al., 2010). Therefore, there are some potential applications in eye-safe laser radar, material processing, laser surgery, remote sensing and effective pump sources as mid-infrared lasers and optical parametric oscillators (Geng et al., 2011; Geng and Jiang, 2014; Slimen et al., 2019; Wang et al., 2019). Up to now, active ions for 2.0 μm laser have been mainly focused on Tm^3+^ and Ho^3+^ ions arising from ${\text{Tm}}^{3+}{:}^{3}{\text{F}}_{4}\to {\;}^{3}{\text{H}}_{6}$ and ${\text{Ho}}^{3+}{:}^{5}{\text{I}}_{7}\to {\;}^{5}{\text{I}}_{8}$ transition. Compared with Ho^3+^, Tm^3+^ owns very strong absorption band of H36→H34 transition and thus can be effectively pumped by commercial high-power 808 nm laser diode. Under the pump scheme, a quantum efficiency of 200% can be expected from “two-for-one” cross relaxation process ${(}^{3}{\text{H}}_{4}{+}^{3}{\text{H}}_{6}\to 2^{3}{\text{F}}_{4}$) (Richards et al., 2010). In addition, broad emission bandwidth of ${\text{Tm}}^{3+}{:}^{3}{\text{F}}_{4}\to^{3}{\text{H}}_{6}$ transition about 300 nm is advantageous to the generation of femtosecond pulse (Agger et al., 2004).

In pursuit of efficient 2.0 μm laser, different glass hosts have been extensively investigated and the laser operation has been demonstrated in silicate, fluoride, germanate and tellurite glasses (Richards et al., 2010; He et al., 2013; Wang et al., 2019). Among these glass hosts, tellurite glasses own a lot of advantage such as broad infrared transmission region, lower phonon energy, high rare-earth ion solubility, high refractive index (~2) and easy fabrication with low melting temperature(Richards et al., 2010). Recently, our groups have exploited several new tellurite glass systems such as TeO_2_-Ga_2_O_3_-BaO (TGB) and TeO_2_-Ga_2_O_3_-ZnO (TGZ) with excellent glass-forming ability, thermal stability and 2.0 μm spectroscopic properties (Li et al., 2019; Mao et al., 2020). To further improve 2.0 μm emission properties, it is very essential to reduce the hydroxyl content in glasses because OH^−^ groups are the main energy loss channels for active ions and can result in strong 2.0 μm fluorescence quenching (Terra et al., 2006). We found that the strength of interaction between Tm^3+^ and OH^−^ (12.9 × 10^−19^ cm^4^/s) was stronger than that between Er^3+^ and OH^−^ (1.9 × 10^−19^ cm^4^/s) (Yuan et al., 2014).

Herein, based on the composition of TGB glass with good thermal stability, we systematically investigate the effects of substitution of BaF_2_ for BaO on physical properties and 1.8 μm emission properties. Density, refractive index, Raman spectra, absorption spectra and emission spectra were measured along with the lifetime of Tm^3+^:^3^F_4_ energy level. Moreover, energy transfer mechanism is proposed and emission cross section and gain coefficient of ${\text{Tm}}^{3+}{:}^{3}{\text{F}}_{4}\to^{3}{\text{H}}_{6}$ transition in TGB glass with 9 mol.% BaF_2_ are determined.

## Materials and Methods

Tm^3+^ doped gallium tellurite glasses (TGB) with the molar compositions of 80TeO_2_-10Ga_2_O_3_-(9-x)BaO-xBaF_2_-1Tm_2_O_3_ (x = 0, 3, 6, and 9) were prepared by the conventional melt-quenching method. TeO_2_, Ga_2_O_3_, BaO, BaF_2_ and Tm_2_O_3_ with 99.99% purity (Aladdin) were used as raw chemicals. Appropriate amounts of these chemicals (~20 g) were well mixed and then melted in an alumina crucible with an alumina lid at ~950°C for 30 min. Afterwards, the melts were poured onto a preheated graphite mold and further annealed at 330°C for 2 h, after which they were cooled slowly inside the furnace to room temperature. The annealed samples for the optical property measurements need to be double-sided polishing into 10 × 10 × 1.5 mm^3^ cylinders. Densities of glasses were determined by the Archimedes' principle using the distilled water as the medium. The refractive index of all the samples was measured by the prism coupling method (Metricon Model 2010) at 633, 1,309, and 1,533 nm with an error of ±5 × 10^−4^. The infrared transmittance spectra were obtained using Vector 33 Fourier transform infrared (FTIR) spectrophotometer (Bruker, Switzerland). The Raman spectra were measured by Raman spectrometer (Renishawin Via, Gloucestershire, UK) and 532 nm laser as the excitation source. Optical absorption spectra measurements were performed on a Perkin-Elmer Lambda 900/UV/VIS/NIR spectrophotometer. The fluorescence spectra were recorded by a computer-controlled Triax 320 type spectrofluorimeter (Jobin-Yvon Corp.) equipped with an InAs detector upon the excitation of an 808 nm LD. After exciting the samples with an 808 nm LD, InAs detector was used to detect the lifetime of Tm^3+^:^3^F_4_ energy level (1.8 μm) along with a digital phosphor oscilloscope (TDS3012C, Tektronix, America) and signal generator. All of the measurements were carried out at room temperature.

## Results and Discussion

**Figure 1:**
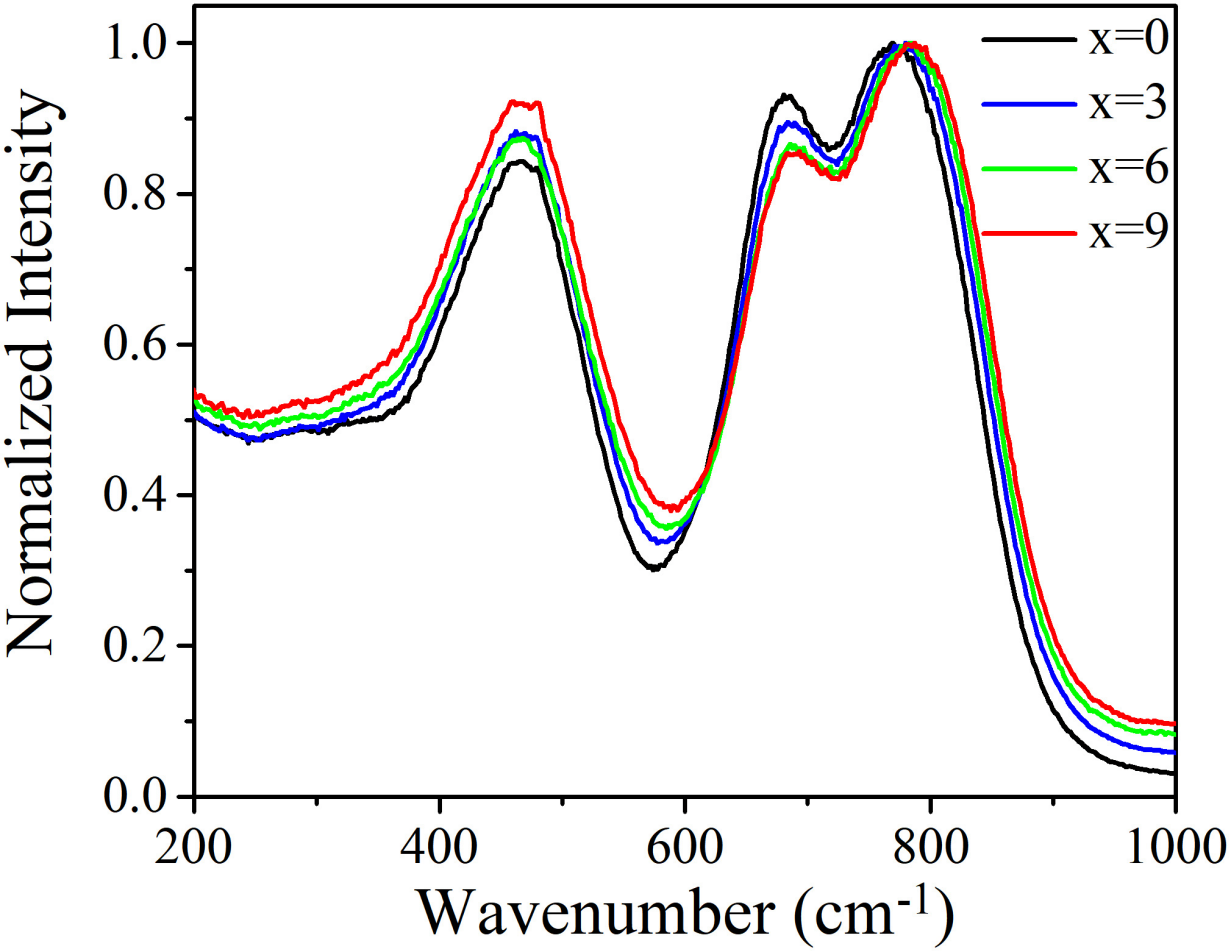
Normalized Raman spectra of TGB glasses with different BaF_2_ amounts.

Table 1 presents the refractive index (*n*) and density (ρ) of TGB glasses with different BaF_2_ contents. It is found that the refractive index and density monotonously decrease when BaF_2_ content increases from 0 to 9 mol.% in step of 3 mol.%. This indicates that the addition of BaF_2_ makes glass network looser (Yang et al., 2017), which is demonstrated by the Raman spectra as shown Figure 1. It is noted that three major bands appear in TGB glasses with different BaF_2_ amounts. The peak A at ~466 cm^−1^ is assigned to the symmetrical stretching or bending vibrations of Te-O-Te linkages at corner sharing sites (Murugan and Ohishi, 2004; Jose et al., 2007). The peak B at ~682 cm^−1^ is ascribed to the anti-symmetric stretching vibrations of Te-O-Te linkages constructed by two un-equivalent Te-O bonds containing bridging oxygens (BO) in TeO_4_ trigonal bipyramid and the peak C is due to the symmetrical stretching vibrations of Te-O^−^ and Te=O bonds with non-bridging oxygens (NBO) in TeO_3_ trigonal pyramid and TeO_3+1_ polyhedra (Murugan and Ohishi, 2004; Jose et al., 2007). It is worth noting that the position of peak C slightly shifts from 769 to 787 cm^−1^ and normalized intensity of peak B declines with the increment of BaF_2_ from 0 to 9 mol.%, revealing that glass network structure is broken and more non-bridging oxygens arise. Such low phonon energy of TGB glasses is able to effectively decrease non-radiative relaxation in favor of the enhancement of 2.0 μm emission intensity.

**Table 1:** The refractive index and density of TGB glasses with different BaF_2_ contents.

**Sample**	***n* (@633 nm)**	***n* (@1,309 nm)**	***n* (@1,533 nm)**	**ρ (g/cm^**3**^)**
x = 0	1.9723	1.9324	1.9289	5.265
x = 3	1.9490	1.9097	1.908	5.219
x = 6	1.9281	1.8920	1.8904	5.140
x = 9	1.9132	1.8792	1.8769	5.128

**Figure 2:**
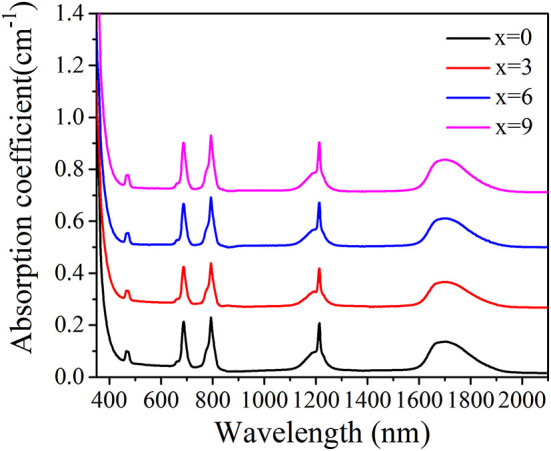
Absorption spectra in TGB glasses with different BaF_2_ contents.

Figure 2 shows the typical absorption spectra of TGB glasses in the wavelength range from 350 to 2,100 nm. The absorption spectrum consists of five absorption bands of Tm^3+^ centered at 473, 687, 794, 1,214, and 1,700 nm, corresponding to respective transitions from the ^3^H_6_ ground state to excited states ^1^G_4_, ^3^F_2_,_3_, ^3^H_4_, ^3^H_5_, and ^3^F_4_. Energy levels above ^1^G_4_ energy level are not clearly identified because of strong intrinsic bandgap absorption in the host glass. It is also found that the position and shape of five absorption peaks are almost constant with the addition of BaF_2_.

**Figure 3:**
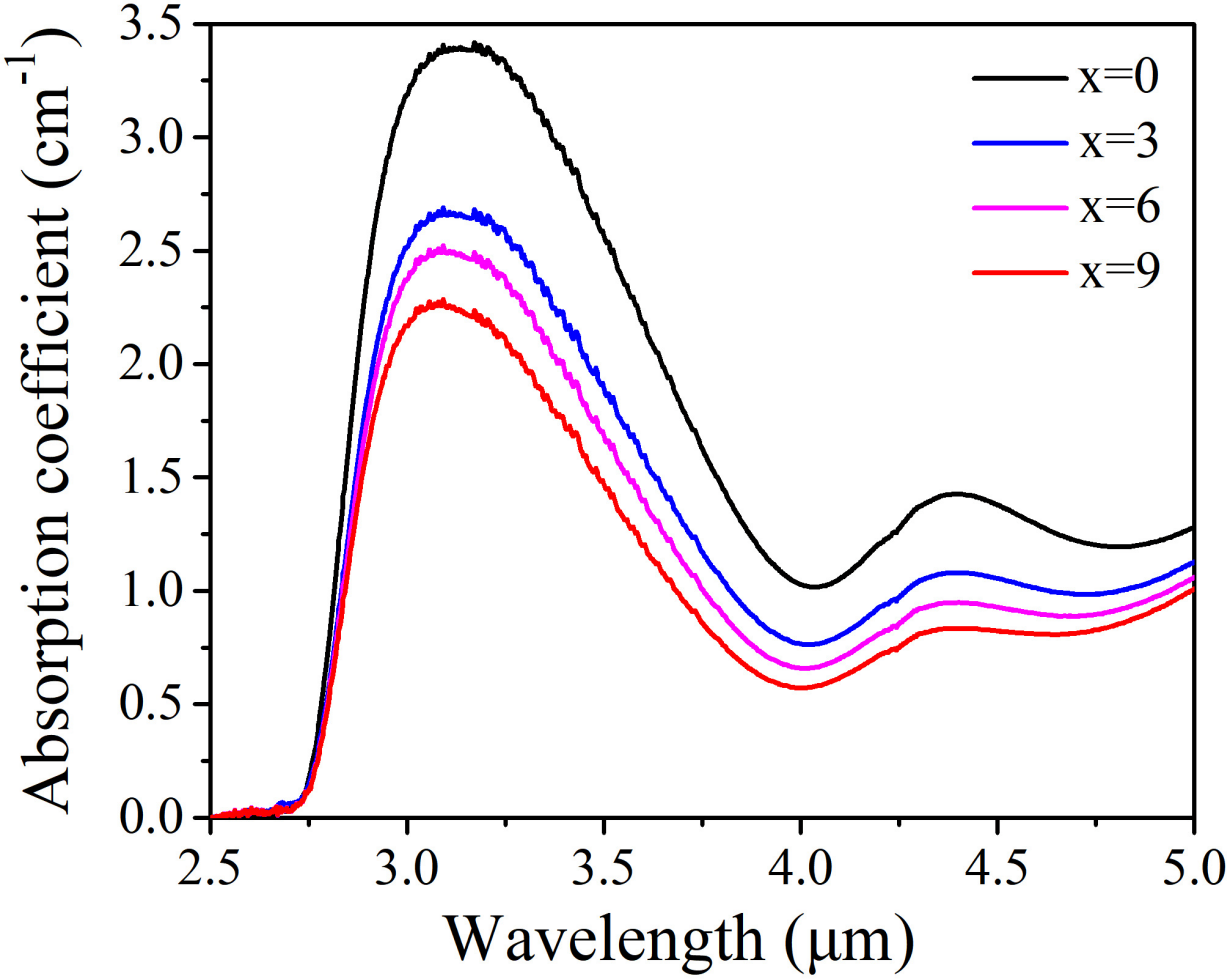
FTIR spectra of TGB glasses with different proportions of BaF_2_.

When BaF_2_ is added, F^−^ ions crack O-H bond in glass network and produce HF gas so that OH^−^ content is reduced. OH^−^ content is reflected by OH^−^ absorption coefficient (α_OH_) (Wang et al., 2013).

(1) $$\begin{array}{l} \alpha_{O\text{H}}=\frac{\text{ln}\left(T_{0}/T\right)}{l} \end{array}$$

where *l* represents the thickness of glass samples, T_0_ and T are the incident and transmitted intensity, respectively. According to FTIR spectra, OH^−^ absorption coefficient of TGB glasses is determined and presented in Figure 3. There are two absorption bands centered at 3.1 and 4.4 μm, corresponding to stretching mode of free Te-OH groups and/or stretching mode of molecular water and stretching mode of strong hydrogen-bonded Te-OH groups, respectively (Wang et al., 2019). α_OH_ at 3.1 μm is obviously higher than the value at 4.4 μm. Moreover, α_OH_ monotonically decreases from 3.4 to 2.2 cm^−1^ with increasing BaF_2_ content from 0 to 9 mol.% in step of 3 mol.%, which is beneficial to improve 1.8 μm emission properties of Tm^3+^ ions.

**Figure 4:**
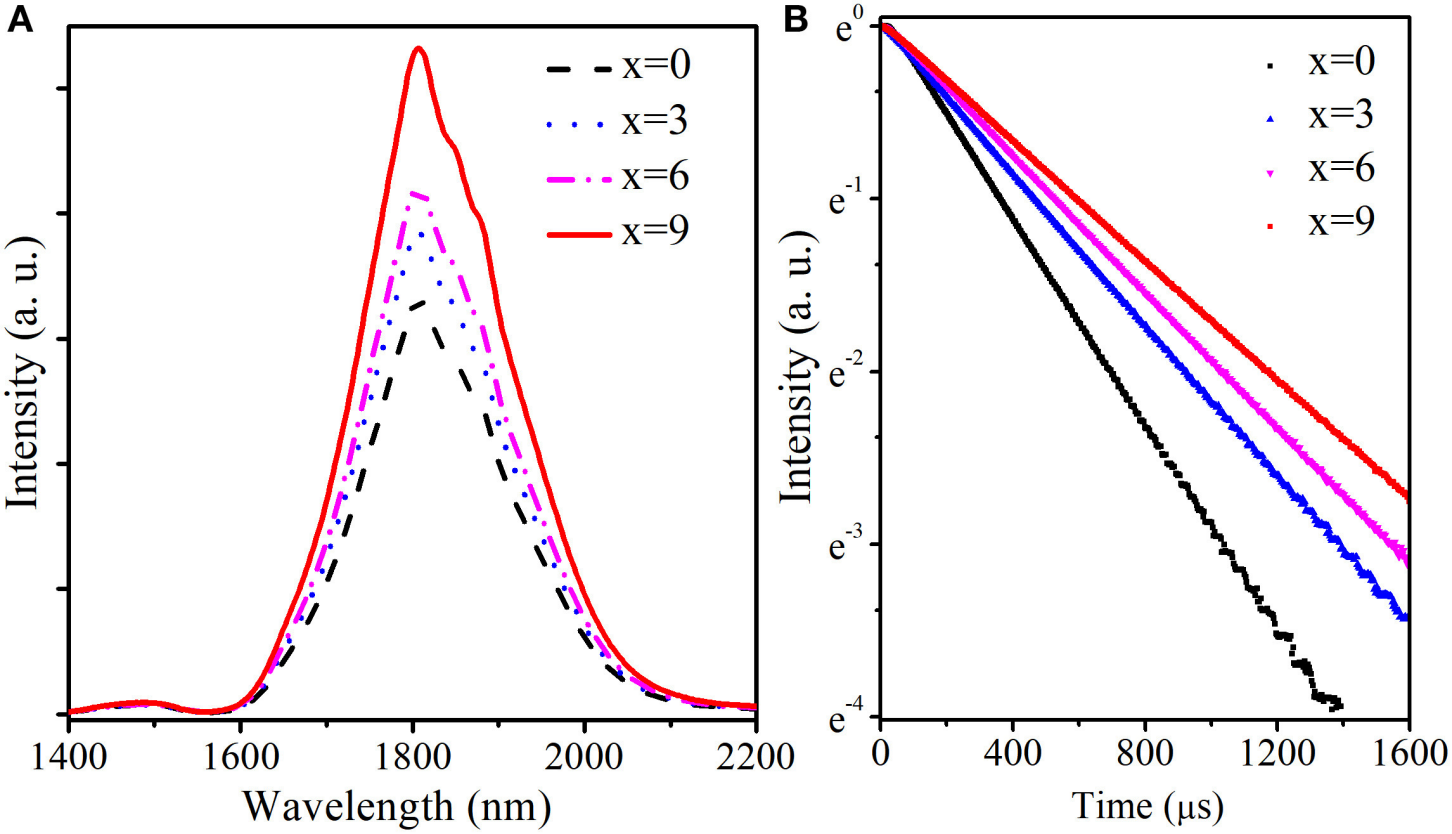
**(A)** Fluorescence spectra and **(B)** decay curves of Tm^3+^:^3^F_4_ energy level in TGB glasses with different proportions of BaF_2_ pumped by 808 nm LD.

Figure 4 compares the fluorescence spectra and decay curves of ${\text{Tm}}^{3+}{:}^{3}{\text{F}}_{4}\to^{3}{\text{H}}_{6}$ transition in TGB glasses with different BaF_2_ amounts pumped by 808 nm LD. From Figure 4A, it is clear that the spectra are characterized by two emission peaks located at 1,488 and 1,808 nm, corresponding to H34→F34 and F34→H36 transitions, respectively. Emission intensity at 1,488 nm is obviously weaker than that at 1,808 nm, which is attributed to effective cross relaxation process ${(}^{3}{\text{H}}_{4}{+}^{3}{\text{H}}_{6}\to 2^{3}{\text{F}}_{4}$). Moreover, emission intensity at 1,488 nm remains almost unchanged and that near 1.8 μm gradually increases with the increment of BaF_2_ concentration. The peak value near 1.8 μm in TGB glasses with 9 mol.% BaF_2_ is 1.6 times as high as that without BaF_2_ because the reduction of OH^−^ content weakens the interaction between Tm^3+^ and OH^−^ and thus enhances radiative transition probability of F34→H36 transition. Figure 4B describes fluorescence decay curves of Tm^3+^:^3^F_4_ energy level monitored at 1,808 nm in TGB glasses with different proportions of BaF_2_. It is clearly noted that the lifetime of ^3^F_4_ energy level gradually prolongs from 337.4 to 577.8 μs when BaF_2_ content increases from 0 to 9 mol.% in step of 3 mol.%. The lifetime in TGB glass with 9 mol.% BaF_2_ is 1.7 times as long as the value without BaF_2_. These results mean that the addition of BaF_2_ can greatly improve 1.8 μm emission properties.

**Figure 5:**
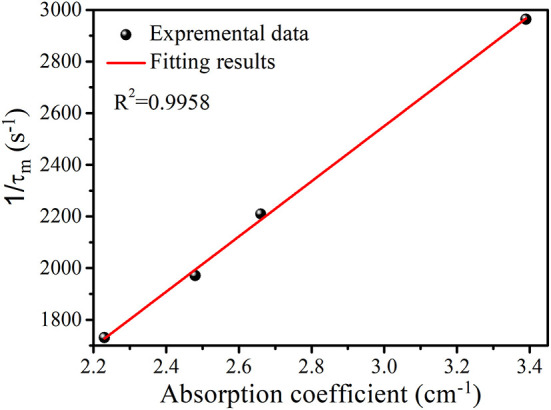
The dependence of the total decay rate on α_OH_ along with the red fitting curve.

In general, the total decay rate (W) of Tm^3+^:^3^F_4_ energy level is defined as the reciprocal of the measured decay lifetime (τ_m_) and is described by the following equations (Zhou et al., 2010).

(2) $$\begin{array}{l} W=1/\tau_{\text{m}}=A_{\text{r}}+W_{OH}+W_{MP}+W_{ET} \end{array}$$

(3) $$\begin{array}{l} W_{OH}=k_{OH-Tm}N_{Tm}\alpha_{OH} \end{array}$$

where A_r_ represents the radiative decay rate, W_OH_ is the energy transfer rate between Tm^3+^ and OH^−^, W_mp_ is the multiphonon decay rate, W_ET_ is the energy transfer rate between Tm^3+^ ions, N_Tm_ is the total concentration of Tm^3+^ ions and α_OH_ is OH^−^ absorption coefficient. k_OH−Tm_ is defined as the strength of interaction between Tm^3+^ and OH^−^ and doesn't rely on the concentrations of Tm^3+^ and OH^−^. Figure 5 represents a good linear relationship between the total decay rate and α_OH_. From this fit, k_OH−Tm_ is determined and equals to 2.82 × 10^−18^ cm^4^/s, which is larger than k_OH−Er_ (1.9 × 10^−19^ cm^4^/s) (Zhou et al., 2010) and lower than k_OH−Tm_ (7.89 × 10^−18^ cm^4^/s) in germanate glasses (Wang et al., 2014).

**Figure 6:**
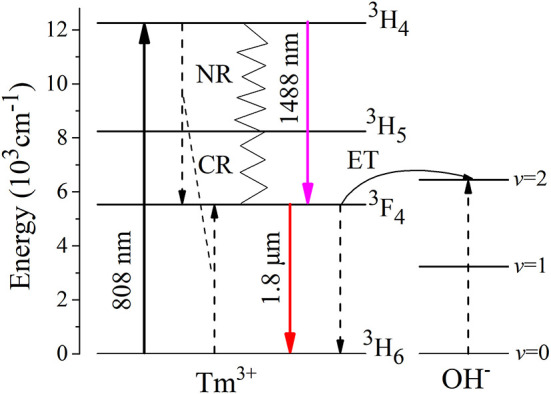
The energy level diagram and energy transfer mechanism of Tm^3+^ ions.

Based on above-mentioned results, Figure 6 shows energy transfer mechanism. Under excitation at 808 nm LD, Tm^3+^ ions are motivated to ^3^H_4_ state from the ^3^H_6_ ground state. Then, a few Tm^3+^ ions return radiatively to ^3^F_4_ state with 1,488 nm photon. However, the majority of ions relax nonradiatively to ^3^F_4_ state via muliphonon relaxation process and efficient cross relaxation process (CR) between two adjacent Tm^3+^ ions ${(}^{3}{\text{H}}_{4}+{\;}^{3}{\text{H}}_{6}\to 2^{3}{\text{F}}_{4}$). Finally, Tm^3+^ ions in the excited ^3^F_4_ state return to the ^3^H_6_ ground state, emitting fluorescence at 1.8 μm. Significantly, the residual OH^−^ in TGB glasses can impair 1.8 μm emission via two OH^−^ ions, indicating that it is essential to decrease the hydroxyl content for improving 1.8 μm emission.

**Figure 7:**
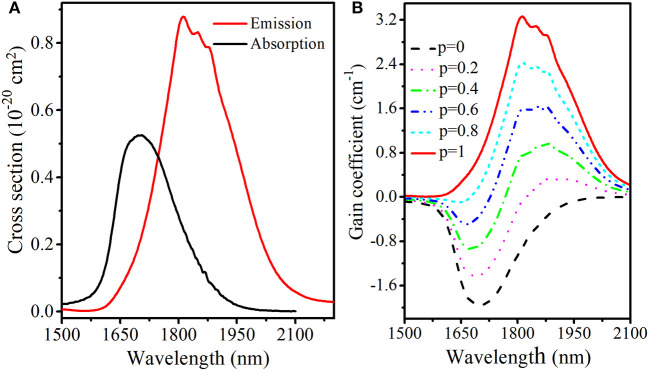
**(A)** Absorption, emission cross section and **(B)** calculated gain coefficient of TGB glass with 9 mol.% BaF_2_.

Both absorption and emission cross sections of Tm^3+^ ions are very crucial parameters to evaluate the potential of TGB glasses as 2 μm laser material. Based on the Beer-Lambert equation and Fuchtbauer-Ladenburg equation (Chen et al., 2007), absorption and emission cross sections of ${\text{Tm}}^{3+}{:}^{3}{\text{H}}_{6}↔{\;}^{3}{\text{F}}_{4}$ transition in TGB glass with 9 mol.% BaF_2_ are calculated and presented in Figure 7A. The maximum absorption cross section of Tm^3+^ reaches 5.3 × 10^−21^ cm^2^ at 1,706 nm, which is higher than that of silicate glass (1.5 × 10^−21^ cm^2^) (Li et al., 2012), fluorophosphate glass (3.0 × 10^−21^ cm^2^) (Li et al., 2015), tellurium germanate glass (3.2 × 10^−21^ cm^2^) (Gao et al., 2015) and germanate glass (4.1 × 10^−21^ cm^2^) (Yu et al., 2009). Moreover, corresponding maximum emission cross section is 8.8 × 10^−21^ cm^2^ at 1,814 nm, which is higher than that of silicate glass (3.6 × 10^−21^ cm^2^) (Li et al., 2012), fluorophosphate glass (5.5 × 10^−21^ cm^2^) (Li et al., 2015), tellurium germanate glass (6.8 × 10^−21^ cm^2^) (Gao et al., 2015), germanate glass (5.5 × 10^−21^ cm^2^) (Yu et al., 2009) and zinc tellurite glass (7.3 × 10^−21^ cm^2^) (Yuan and Xiao, 2018). The high emission cross section of TGB glass with 9 mol.% BaF_2_ is helpful to provide high laser gain.

Once absorption and emission cross sections are determined and it is supposed that Tm^3+^ ions are only in either the ^3^H_6_ or ^3^F_4_ state, the gain coefficient *G(*λ*)* of Tm^3+^ near 1.8 μm can be obtained by the following equation (Zou and Toratani, 1996).

(4) $$\begin{array}{l} G\left(\lambda \right)=N\left[p\sigma_{e}-\left(1-p\right)\sigma_{a}\right] \end{array}$$

where *N* represents the total concentration of Tm^3+^ ions and *p* is the inversion factor given by the ratio between the population of lasing upper level (^3^F_4_) and the total concentration that ranges from 0 to 1. Figure 7B shows gain coefficient spectrum of TGB glass with 9 mol.% BaF_2_. It is found that the gain peak shifts to shorter wavelength with increasing *p*, which is a typical feature of the quasi-three-level system. Moreover, gain coefficient starts to be greater than zero in the wavelength range from 1,824 to 2,100 nm when *p* ≥ 0.2 and the maximum value is 3.3 cm^−1^ at 1,814 nm, which is larger than that of silicate glass (2.57 cm^−1^) (Tang et al., 2016), germanate glass (2.11 cm^−1^) (Slimen et al., 2019) and tellurite glass (0.91 cm^−1^) (Tian et al., 2019). This means that TGB glass with 9 mol.% BaF_2_ is a promising host material for efficient 2.0 μm fiber laser development.

## Conclusions

In summary, the effects of BaF_2_ contents on density, refractive index, Raman spectra, OH^−^ absorption coefficient and 1.8 μm spectroscopic properties of Tm^3+^ doped gallium tellurite glasses are studied in detail. When BaF_2_ content increases from 0 to 9 mol.% in step of 3 mol.%, refractive index and density gradually reduce. Meanwhile, α_OH_ monotonically decreases from 3.4 to 2.2 cm^−1^, which makes emission peak value near 1.8 μm in TGB glass with 9 mol.% BaF_2_ being 1.6 times as large as that without BaF_2_ while the lifetime becomes 1.7 times as long as the value without BaF_2_. The maximum emission cross section at around 1.8 μm of TGB glass with 9 mol.% BaF_2_ reaches 8.8 × 10^−21^ cm^2^. In addition, positive gain coefficient in the wavelength range from 1,824 to 2,100 nm is achieved when *p* ≥ 0.2 and the maximum gain coefficient is 3.3 cm^−1^ at 1,814 nm. As a result, TGB glass with 9 mol.% BaF_2_ appears to be a highly promising host material for efficient 2.0 μm fiber laser development.

## Data Availability Statement

The raw data supporting the conclusions of this article will be made available by the authors, without undue reservation.

## Author Contributions

JY, PX, and WW conceived the idea. JY and PX wrote the paper. TD, YY, DO, JC, and SY advised the paper. All authors contributed to the article and approved the submitted version.

## Conflict of Interest

The authors declare that the research was conducted in the absence of any commercial or financial relationships that could be construed as a potential conflict of interest.
